# Presumed *COL4A3/COL4A4* Missense/Synonymous Variants Induce Aberrant Splicing

**DOI:** 10.3389/fmed.2022.838983

**Published:** 2022-03-21

**Authors:** Haiyue Deng, Yanqin Zhang, Jie Ding, Fang Wang

**Affiliations:** Department of Pediatrics, Peking University First Hospital, Beijing, China

**Keywords:** *COL4A3*, *COL4A4*, splicing variant, minigene, genetic counselling

## Abstract

**Background:**

The incorrect interpretation of missense and synonymous variants can lead to improper molecular diagnosis and subsequent faulty genetic counselling. The aim of this study was to evaluate the pathogenicity of presumed *COL4A3/COL4A4* missense and synonymous variants detected by next-generation sequencing to provide evidence for diagnosis and genetic counselling.

**Methods:**

Patients' clinical findings and genetic data were analysed retrospectively. An *in vitro* minigene assay was conducted to assess the effect of presumed *COL4A3/COL4A4* missense and synonymous variants on RNA splicing.

**Results:**

Five unclassified *COL4A3/COL4A4* variants, which were detected in five of 343 patients with hereditary kidney diseases, were analysed. All of them were predicted to affect splicing by Human Splicing Finder. The presumed *COL4A3* missense variant c.4793T > G [p. (Leu1598Arg)] resulted in a loss of alternative full-length transcript during the splicing process. The *COL4A3* transcript carried synonymous variant c.765G > A [p. (Thr255Thr)], led to an in-frame deletion of exon 13. Nevertheless, variants c.3566G > A [p. (Gly1189Glu)] in *COL4A3* and c.3990G > A [p. (Pro1330Pro)], c.4766C > T [p. (Pro1589Leu)] in *COL4A4* exhibited no deleterious effect on splicing. Among the five patients harbouring the abovementioned *COL4A3/COL4A4* variants, three patients were genetically diagnosed with autosomal recessive Alport syndrome, one patient was highly suspected of having thin basement membrane nephropathy, and the other patient was clinically diagnosed with Alport syndrome.

**Conclusions:**

*COL4A3* presumed missense variant p. (Leu1598Arg) and synonymous variant p. (Thr255Thr) affect RNA splicing, which highlights the prime importance of transcript analysis of unclassified exonic sequence variants for better molecular diagnosis and genetic counselling. Meanwhile, the reliability of splicing predictions by predictive tools for exonic substitutions needs to be improved.

## Introduction

The widespread application of next-generation sequencing (NGS) in clinical settings is leading to incredible progress in the molecular diagnostics of human inherited disorders. However, a large number of unknown significance or unclassified variants in disease genes pose a challenge for genetic diagnosis and subsequent genetic counselling. For example, *COL4A3/COL4A4* are the causative genes of both autosomal inherited Alport syndrome and thin basement membrane nephropathy (1), which are chronic haematuria nephropathies with significantly different prognoses. Currently, 22% of the reported *COL4A3/COL4A4* variants are classified as uncertain significance, and most of them are missense variants (refer to ClinVar database, https://www.ncbi.nlm.nih.gov/clinvar). It is difficult to assess such variants as pathogenic or neutral based solely on genomic analysis.

A study showed (2) that 5–10% of human genes contain at least one region where synonymous variants could be harmful. Some missense and synonymous variants could interfere with protein expression, conformation or function by affecting precursor mRNA (pre-mRNA) splicing, although they may only change one amino acid or not at genomic DNA level (3, 4). Our previous study demonstrated that 6 presumed missense variants caused splicing defects by analysing *COL4A5* mRNA in skin fibroblasts (5). Therefore, transcript analysis to demonstrate the effect of unknown significance variants on pre-mRNA splicing is crucial during the assessment of their pathogenicity.

In recent years, a variety of bioinformatics tools have been developed and applied to predict their effects on pre-mRNA splicing. Nevertheless, the predicted *in silico* results may not necessarily match the transcription process *in vivo* (6), and functional experiments are still needed. Our previous study increased the detection rate of *COL4A5* splicing mutations to 25% by analysing the cDNA of skin fibroblasts (17.6% on average) (7). However, since *COL4A3/COL4A4*-encoding proteins are expressed in the kidney, cochlea, and eyes, it is difficult to obtain appropriate material from patients for transcript analysis. A minigene assay *in vitro*, an attractive alternative method to assess the impact of unclassified variants on splicing, is a simple and useful solution to overcome the unavailability of patient-derived specimens.

In this study, we used a minigene assay to evaluate the pathogenicity of five presumed missense and synonymous variants in *COL4A3/COL4A4* detected by NGS to provide evidence for diagnosis and genetic counselling.

## Materials and Methods

The clinical data and gene testing results of all patients in this study were retrospectively collected from the online hereditary renal diseases registry database established in 2012 by Peking University First Hospital, the procedures were approved by the Ethical Committee of Peking University First Hospital (approval number: 2016[1179]), and informed consent was obtained from the participants or their parents.

### Patients

Patients who were registered in the online registry of hereditary kidney diseases, admitted to our hospital from February 2015 to September 2018 and met the inclusion and exclusion criteria were screened in our study. The inclusion criteria were as follows: (1) haematuria; (2) missense or synonymous variants in *COL4A3/COL4A4* detected by NGS; (3) extremely rare variants with minor allele frequency < 0.01% (8); (4) variants in a highly conserved amino acid of the protein; (5) harmful variants predicted by bioinformatics tools (Mutation Taster/SIFT/Polyphen-2/GERP++_RS/CADD_phred); and (6) Human Splicing Finder predicted that the variants affected splicing. We excluded patients detected with *COL4A3/COL4A4* pathogenic/likely pathogenic variants evaluated by American College of Medical Genetics and Genomics (ACMG) guidelines or those with unavailable medical records.

### Research Methods

#### Clinical Data and Genotypes

Patient information, including sex, age, height, weight, family history, clinical findings (age at onset of disease, renal and extrarenal features), skin and renal biopsy results, and genotypes, was reviewed.

All *COL4A3/COL4A4* variants were verified by Sanger sequencing, and direct sequencing of corresponding variants was performed if parental genomic DNA samples were available. Meanwhile, the effect on RNA splicing of these variants was assessed using another three available splicing prediction tools including NNSPLICE (http://www.fruitfly.org/seq_tools/splice.html), NetGene2 (http://www.cbs.dtu.dk/services/NetGene2/), and MaxEntScan (http://hollywood.mit.edu/burgelab/maxent/Xmaxentscan_scoreseq.html). NNSPLICE and NetGene2 present scores of 0–1 for the predicted site; the higher the score the more likely a variant is a splicing site.

#### Nomenclature

All genetic sequences involved in this study were referred to the UCSC Genome Browser and human GRCh37/hg19 (http://genome.ucsc.edu/). The DNA sequence numbering was based on the cDNA sequence for *COL4A3* (NM_000091.5)/*COL4A4* (NM_000092.5), following the recommendations of the Human Genome Variation Society (HGVS, c.1 represented the first position of the translation initiation codon).

#### Minigene Assay

##### Generation of Minigene Constructs

The assay relied on the use of a minigene vector, which contained a fragment of *COL4A3*/*COL4A4*. The genomic segment encompassing the variant exon sequence and at least one exon nearby it, along with 150 bp of the corresponding flanking intronic sequence, were inserted into the minigene expression plasmid pcDNA3.1 (ViGene Biosciences, Shandong, China). Two minigene constructs (wild-type and mutant-type) were built, and the sequence was verified by direct sequencing after synthesis.

##### Cell Culture and Transfection

HEK293T cells were cultured in DMEM–GlutaMAX (Gibco, New York, USA) containing 10% foetal bovine serum (Gibco, New York, USA) and 1% penicillin–streptomycin amphotericin (Gibco, New York, USA). The wild-type and mutant minigene constructs were transiently transfected into HEK293T cells using jetPRIME (Polyplus, Strasbourg, France) according to the manufacturer's instructions. Cells were harvested 24 h post-transfection.

##### RNA Isolation and Reverse Transcription-Polymerase Chain Reaction (RT–PCR) Analysis

Total RNA was extracted from the transfected cells using TRIzol reagent (Invitrogen, California, USA) according to the manufacturer's instructions. The RT experiment was performed using TransScript One-Step gDNA Removal and cDNA Synthesis SuperMix (TransGen Biotech, Beijing, China). The RT–PCR thermal profile was as follows: 25°C for 10 min, 42°C for 30 min, and 85°C for 5 s.

##### PCR Amplification of cDNA

Five pairs of primers were designed to amplify the resulting cDNA by PCR (Table 1). The PCR parameters were optimised as follows: 94°C for 5 min, 35 cycles of denaturation (94°C for 30 s), annealing (58°C for 30 s), and elongation (72°C for 45 s), and a final elongation at 72°C for 7 min. The amplification products were separated by 2% agarose gel electrophoresis and sequenced using a 3730XL automatic sequencer (Applied Biosystems).

**Table 1:** PCR primer sequence.

**Variants**	**Forward primer(5^**′**^-3^**′**^)**	**Reverse primer(5^**′**^-3^**′**^)**
A	CCTGCAGCGATTTACCACAA	TGTGGGGAAACAAATTGAAACTT
B	GGAACCCTGGTGCTCAAG	TCCCATGTCTCCTGCAGTTC
C	CTGGCTAGCGTTTAAACTTAAGC	TTGCTCCCTTGTCTCCCTTT
D	TCCCTGGGAGTGTTGACCT	GGCCCTTTTCTCCCTGGA
E	GTTCTCCACAGTCAGACGGA	CTGGAGCAGAGGAAAACTGC

Splicing defects were determined according to agarose gel image patterns of the amplification products containing the wild-type and variant alleles. The criteria were based on those proposed by Aoto et al. (9).

## Results

According to the inclusion and exclusion criteria, our attention was caught by five *COL4A3/COL4A4* variants detected in five patients from 343 patients with hereditary kidney diseases. These variants were evaluated as uncertain significance by ACMG guidelines. *COL4A3* gene variants c.4793T>G [p. (Leu1598Arg)] (109) and p. (Thr255Thr) (10) have been previously reported, and *COL4A4* variant c.3990G > A [p. (Pro1330Pro)] has been reported in ClinVar, whereas the effect of these three variants on pre-mRNA splicing has not been reported. The splicing effects predicted by *in silico* splice tools are shown in Table 2. Aberrant RNA splicing effects for these five variants were predicted by Human Splicing Finder, and 4 out of the 5 variants were predicted as deleterious by at least another tool.

**Table 2:** Description of splicing effects predicted by *in silico* splice tools.

**Variant**	**Predictors of** ***in silico*** **splice tools**						
	**Human splicing finder**		**NNSPLICE**		**NetGene2**		**MaxEntScan**
	**Acceptor**	**Donor**	**Acceptor**	**Donor**	**Acceptor**	**Donor**	
A: *COL4A3* Exon 51-c.4793T > G, p. (Leu1598Arg)	Alteration of an exonic ESE site		Same score between wild type and variant sequences		Same score between wild type and variant sequences		Same score between wild type and variant sequences
B: *COL4A3* Exon 42-c.3566G > A, p. (Gly1189Glu)	Creation of an exonic ESS site; Alteration of an exonic ESE site		Same score between wild type and variant sequences		WT: 0.07 Mut: 0.22		WT: 0.98 Mut: 0.42
C: *COL4A3* Exon 13-c.765G > A, p. (Thr255Thr)		WT: 83.68 Mut: 73.59		WT: 0.98 Mut: 0.42		WT: 0.51 Mut: 0	WT: −24.93 Mut:−30.37
D: *COL4A4* Exon 42-c.3990G > A, p. (Pro1330Pro)	Activation of an exonic cryptic acceptor site; Creation of an exonic ESS site		Same score between wild type and variant sequences		WT: 0 Mut: 0.42		Same score between wild type and variant sequences
E: *COL4A4* Exon 47-c.4766C > T, p. (Pro1589Leu)	WT: 53.65 Mut: 81.52		WT: 0.63 Mut: 0.66		WT: 0.94 Mut: 0.82		WT: 9.85 Mut: 8.04

*The reference sequence of COL4A3 and COL4A4 transcripts was NM_000091.5 and NM_000092.5, respectively. WT, wild-type sequence; Mut, variant sequence; ESE, exonic splicing enhancer; ESS, exonic splicing silencer*.

### Characterisation of Phenotypes and Genotypes

**Figure 1:**
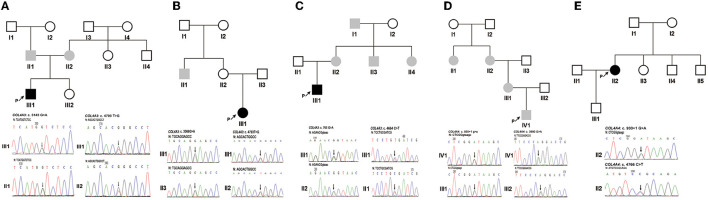
The pedigree of five families and Sanger sequencing of PCR amplified products containing the variants for probands 1–5 and their parents. **(A)** Family 1. **(B)** Family 2. **(C)** Family 3. **(D)** Family 4. **(E)** Family 5. Black arrows indicate the probands and the changed nucleotides. The filled black squares and circles indicate the individuals who presented with haematuria and proteinuria, and the filled grey squares and circles indicate the individuals who featured only haematuria. N, normal sequence. Intron and exon sequences are depicted by lower and capital case letters, respectively. The reference sequence of *COL4A3* and *COL4A4* transcripts was NM_000091.5 and NM_000092.5, respectively.

Patient 1 (III1 of family 1) was a 1.4-year-old boy who visited the hospital due to a darkening urine colour. The laboratory investigation showed haematuria [urinary erythrocytes were full of view under urinary sediment microscopy] and proteinuria [protein-to-creatinine ratio (PCR): 1.55 g/gcr; albumin-to-creatinine ratio (ACR): 295.74 mg/g]. His non-consanguineous parents both had haematuria (Figure 1A). NGS and Sanger sequencing revealed that Patient 1 had compound heterozygous *COL4A3* missense variants [exon 51: c.4793T > G, p. (Leu1598Arg), maternal; exon 37: c.3143G > A, p. (Gly1048Asp), paternal] (Figure 1A). These two variants were both evaluated as uncertain significance by ACMG guidelines. Among them, the allele frequency of c.4793T > G was 0 in the 1000 Genomes Project, Exome Sequencing Project (ESP) database, and the gnomAD South Asian, African/African-American, Lation/Admixed American, Ashkenazi Jewish, European and other database, 0.0007678 in the gnomAD East Asian database, and 0.00005341 in the gnomAD database. Amino acid Leucine (Leu)-1598 is highly conserved in vertebrates. *In silico* predictors such as MutationTaster, SIFT, PolyPhen-2 and CADD_phred all predicted it to be deleterious. Only Human Splicing Finder indicated that it may have an effect on splicing.

Patient 2 (III1 of family 2) presented with oedema, haematuria [20–30 red blood cells per high-power field (RBCs/HPF)], and proteinuria (1+, detected by the dipstick test) when she was 7 years old. Prednisone at a standard dose and mycophenolate mofetil treatment did not improve the proteinuria. Subsequently, renal biopsy performed in the local hospital indicated focal segmental glomerular sclerosis (FSGS) under a light microscope and segmental thickening and a lamellated glomerular basement membrane (GBM) under an electron microscope. The uncle of patient 2 had a history of haematuria for 4–5 years, but his urine protein was negative. Renal biopsy indicated thin basement membrane nephropathy (TBMN). Neither ocular nor hearing abnormalities were found (Figure 1B). Two *COL4A3* missense variants [exon 42: c.3566G > A, p. (Gly1189Glu), paternal; exon 51: c.4793T > G, p. (Leu1598Arg), maternal] (Figure 1B) in a compound heterozygous state were detected in genomic DNA extracted from the peripheral blood leukocytes of patient 2. They were all evaluated as uncertain significance by ACMG guidelines. Among them, the allele frequency of c.3566G > A was 0 in the 1000 Genomes Project, and ESP databases. Amino acid Glycine (Gly)-1189 is highly conserved in vertebrates. MutationTaster, SIFT, PolyPhen-2 and CADD_phred all predicted it to be deleterious. Human Splicing Finder, NetGene2 and MaxEntScan indicated that it may have an effect on splicing.

Patient 3 (III1 of family 3) was a 25-year-old man. Haematuria (10–20 RBCs/HPF) and proteinuria (1+) were detected when he was 3 years old. His renal function was normal, whereas at the age of 23 years old, he developed gout, and an elevated level of serum creatinine (150 μmol/L) was detected. Renal biopsy revealed mild mesangial proliferative glomerulonephritis under a light microscope and segmental thickening and thinning and lamellated GBM under an electron microscope. He had no ocular or hearing abnormalities. Laboratory findings showed his serum creatinine was 163 μmol/L, urinary protein was 0.76 g/24 h and estimated glomerular filtration rate was 49.6 ml/min/1.73 m^2^ when he was 25 years old. His multiple maternal members (including his mother, aunt, uncle, and grandfather) only presented with haematuria (Figure 1C). *COL4A3* synonymous and missense variants [exon 13: c.765G > A, p. (Thr255Thr) maternal; exon 50: c.4664C > T, p. (Ala1555Val), paternal] (Figure 1C) in a compound heterozygous state were detected in patient 3's peripheral blood leukocytes genomic DNA. They were all evaluated as uncertain significance by ACMG guidelines. Among them, the allele frequency of c.765G > A was 0 in the 1000 Genomes Project, ESP databases, and gnomAD East Asian, South Asian, Lation/Admixed American, Ashkenazi Jewish, European (Finnish) and other database, 0.00004136 in the gnomAD African/African-American database, 0.000007801 in the gnomAD European (non-Finnish) database, and 0.000007133 in the gnomAD database. This variant was in the last nucleotide of exon 13. *In silico* MutationTaster predicted it to be deleterious. Human Splicing Finder, NNSPLICE, NetGene2 and MaxEntScan indicated that it may have an effect on splicing.

Patient 4 (IV1 of family 4) was an 11.3-year-old boy. From the age of 3, multiple laboratory tests showed haematuria, no proteinuria, and normal renal function. Renal biopsy was performed on his grandmother at the local hospital due to positive urine occult blood when she was 53 years old. Her GBM was observed to have irregular thickening under electron microscopy. Her urinary protein was negative, and her renal function was normal. At the age of 50, she was found to have hearing loss and needed to wear a hearing aid. Haematuria without proteinuria was detected in the grandmother's sister when she was 55 years old. She had normal renal function without hearing loss or ocular abnormalities. Patient 4's mother had increased numbers of urinary red blood cells without proteinuria or hearing loss (Figure 1D). *COL4A4* synonymous and splicing variants [exon 42: c.3990G > A, p. (Pro1330Pro), paternal; intron 15: c.930 + 1G > A, maternal] (Figure 1D) in a compound heterozygous state were detected in patient 4's peripheral blood leukocytes genomic DNA. They were evaluated as uncertain significance and pathogenic by ACMG guidelines. Among them, the allele frequency of c.3990G > A was 0 in the 1000 Genomes Project, ESP databases and the gnomAD African/African-American, Lation/Admixed American, Ashkenazi Jewish, European and other database, 0.0006783 in the gnomAD East Asian database, 0.00009902 in the gnomAD South Asian database, and 0.00006097 in the gnomAD database. *In silico* MutationTaster predicted it to be deleterious. Human Splicing Finder and NetGene2 indicated that it may have an effect on splicing.

Patient 5 (II2 of family 5) was a 34-year-old woman who manifested haematuria and proteinuria for seven years. Laboratory investigation showed haematuria (5–10 RBCs/HPF) and proteinuria (ACR: 570.22 mg/g; urinary total protein: 1.04 g/24 h), and the estimated glomerular filtration rate was 81.9 ml/min/1.73 m^2^. Renal biopsy under electron microscopy suggested that the GBM ultrastructural changes were in accordance with Alport syndrome. Haematuria and/or proteinuria were not observed in her family members (Figure 1E). *COL4A4* heterozygous missense and splicing variants [exon 47: c.4766C > T, p. (Pro1589Leu); intron 15: c.930 + 1G > A] (Figure 1E) were detected in patient 5's peripheral blood leukocytes genomic DNA. Lacking DNA samples from her parents, the evaluation of the *cis* vs. *trans* nature of these two variants was not done. They were evaluated as uncertain significance (PM2, PM3, PP3) and pathogenic (PVS1, PM2, PP3) by ACMG guidelines. Among them, the allele frequency of c.4766C > T was 0 in the 1000 Genomes Project, ESP databases and the gnomAD African/African-American, Lation/Admixed American, Ashkenazi Jewish and other database, 0.00005595 in the gnomAD East Asian database, 0.00003272 in the gnomAD South Asian database, 0.00005332 in the gnomAD European (non-Finnish) database, 0.00004716 in the gnomAD European (Finnish) database, and 0.00003629 in the gnomAD database. Amino acid Proline (Pro)-1589 is highly conserved in vertebrates. MutationTaster, SIFT, PolyPhen-2 and CADD_phred all predicted it to be deleterious. Human Splicing Finder and MaxEntScan indicated that it may have an effect on splicing.

### The Splicing Effect of Two Variants in *COL4A3*: c.4793T > G and c.765G > A

**Figure 2:**
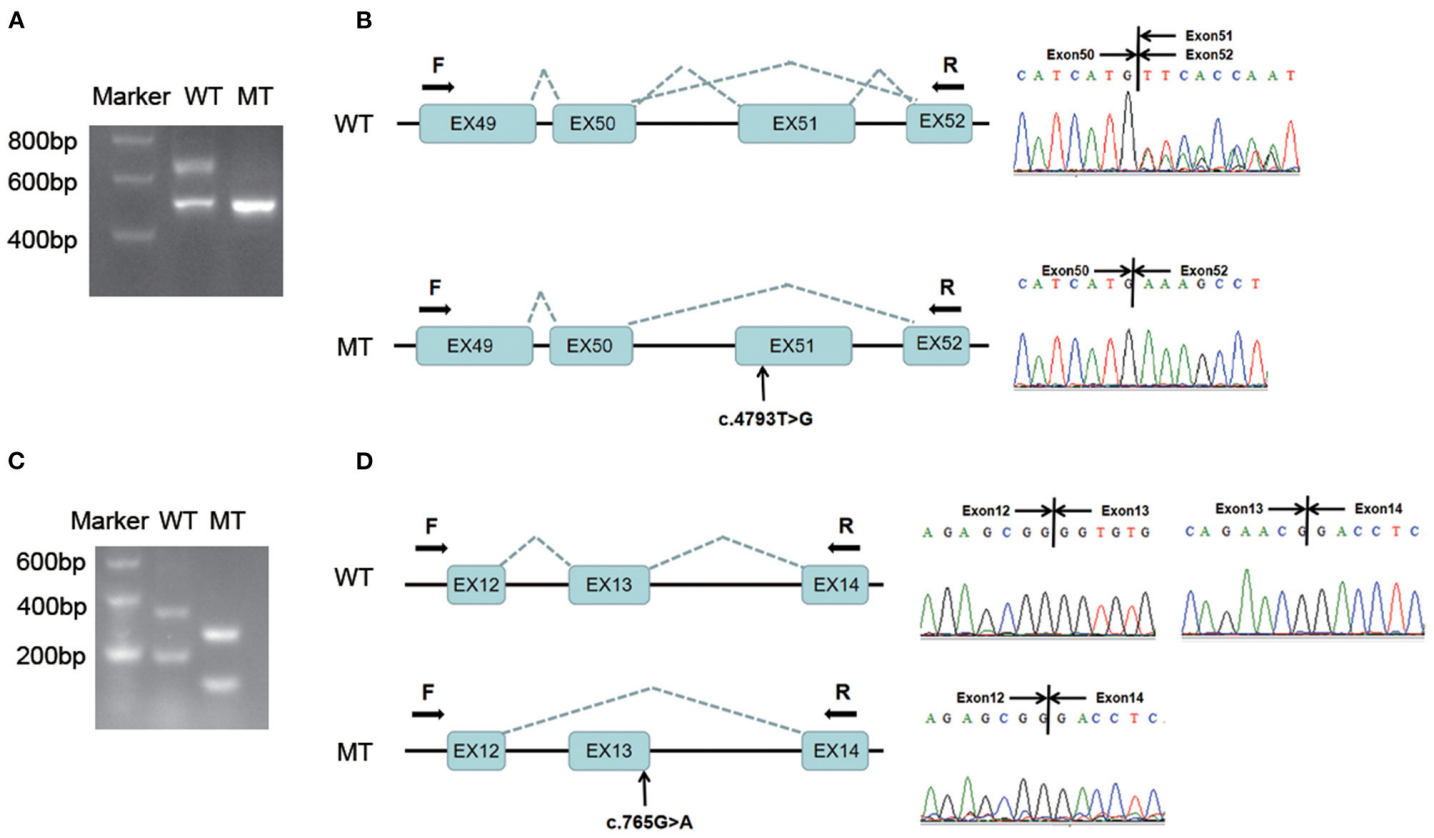
Minigene assay analysis of two variants in COL4A3: c.4793T > G and c.765G > A. **(A)** The amplification products of the wild-type and variant minigene constructs (*COL4A3*: c.4793T > G) were separated by 2% agarose gel electrophoresis. The wild-type transcription product had two bands, while the variant transcription product exhibited only one band after PCR amplification. **(B)** Direct sequencing of the PCR products showed that there were two alternative splicing transcripts exon51+/exon51– in the wild-type minigene constructs, while the variant transcript skipped exon 51 during the splicing process. **(C)** The amplification products of the wild-type and variant minigene constructs (c.765G > A) were separated by 2% agarose gel electrophoresis. Although the wild-type and variant transcription products exhibited two bands after PCR amplification, the variant transcripts were all shorter than those of the wild-type. **(D)** Direct sequencing of the PCR products revealed that the two variant transcripts all skipped exon 13 during the splicing process. WT, wild-type; MT, mutant-type. F, forward primer; R, reverse primer.

Agarose gel electrophoresis showed that the wild-type minigene transcription product had two bands, suggesting alternative splicing, while the variant transcription product (c.4793T > G) exhibited only one band after PCR amplification (Figure 2A). Direct sequencing of the PCR products showed that there were two wild-type transcripts (679 bp and 506 bp long) and a variant transcript (506 bp). The longer wild-type transcript contained exons 49, 50, 51 and 52, while the shorter transcript skipped exon 51 (173 bp); the variant transcript also skipped exon 51 during the splicing process (Figure 2B). These results indicated that the variant c.4793T > G could affect splicing at the mRNA level, which was consistent with the prediction of Human Splicing Finder.

The variant (c.4793T > G) was reclassified according to the ACMG guidelines and the minigene assay results. It could result in a deletion of the normal transcript, which belonged to the null variant (PVS1), and was re-evaluated as “pathogenic” based on the existing evidence (PVS1, PM2, PP3, and PP5). The variant *in trans* c.3143G > A was reclassified as “likely pathogenic” (in line with PM2, PM3, PP3 and PP4). Autosomal recessive Alport syndrome was diagnosed in patient 1 according to his clinical manifestation, family history of haematuria, and genetic testing results. The recurrence risk of his siblings was 25%.

If primers were located at the inserted exons, the variant transcript (c.765G > A) would be <100 bp and could not be sequenced, so we set the forward primer on the plasmid. The wild-type minigene transcription products were all longer than those of the variants (c.765G > A) after PCR amplification and agarose gel electrophoresis (Figure 2C). Direct sequencing of the PCR products showed that the two variant transcripts all skipped exon 13 during the splicing process (Figure 2D), which is predicted to cause an in-frame deletion of 26 amino acids during translation. The results indicated that it affected splicing at the mRNA level, consistent with the prediction of four splice site prediction tools.

The variant c.765G > A was reclassified as “pathogenic” (PVS1, PS3, PM2, PP3) according to the ACMG guidelines and minigene assay results. The variant c.4664C > T *in trans* was classified as likely pathogenic (PM2, PM3, PP3, PP4). Autosomal recessive Alport syndrome could be diagnosed in patient 3. The recurrence risk of his siblings was 25%.

### Variants c.3566G > A in *COL4A3* and c.3990G > A and c.4766C > T in *COL4A4* Exhibited no Deleterious Effect on Splicing

**Figure 3:**
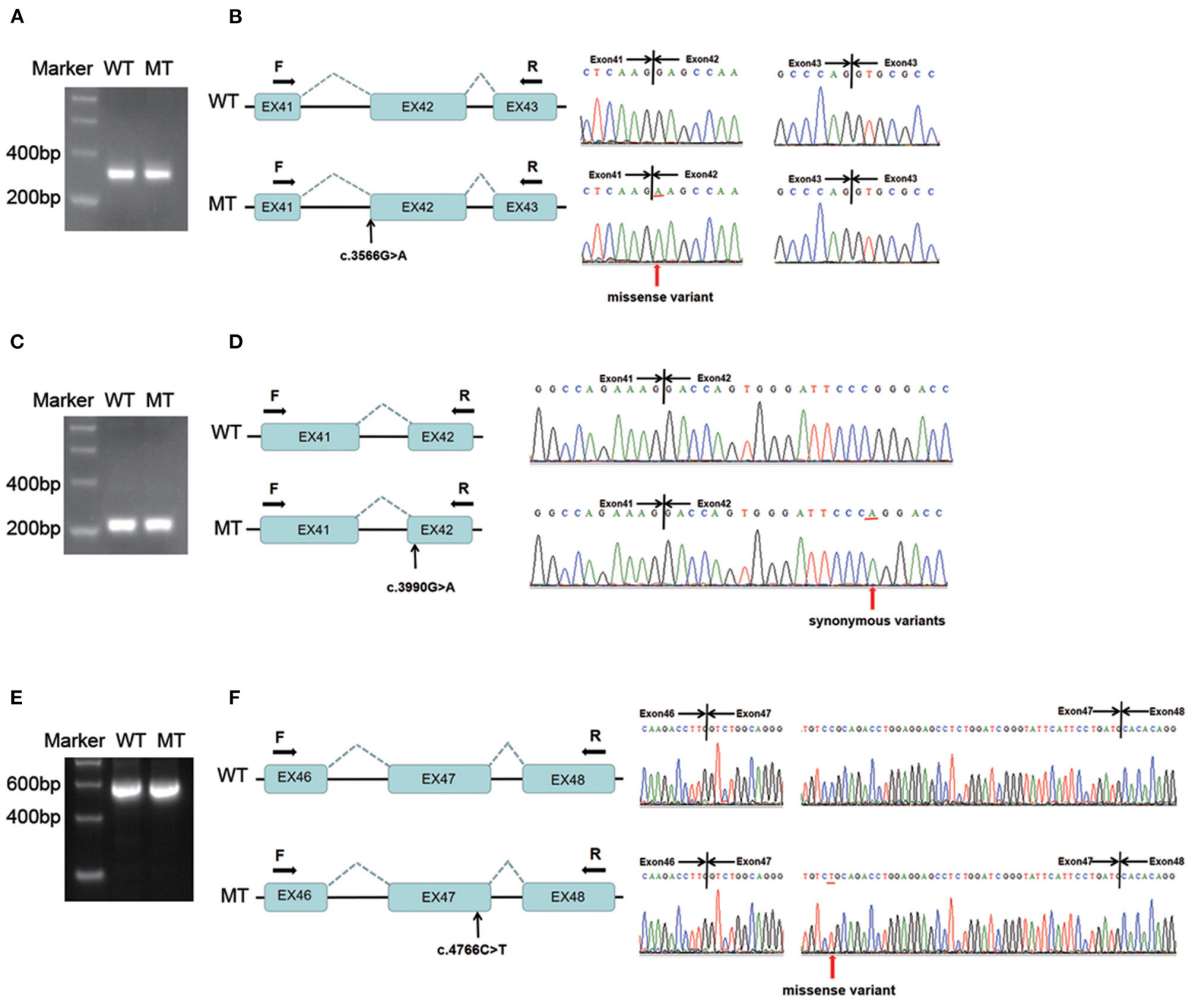
Minigene assay analysis of variants c.3566G > A in *COL4A3* and c.3990G > A, c.4766C > T in *COL4A4*. **(A)** The amplification products of the wild-type and variant minigene constructs (c.3566G > A) were separated by 2% agarose gel electrophoresis. The lengths of the two bands were the same. **(B)** Direct sequencing showed that the two transcripts were identical except for the mutant nucleotide (c.3566G > A). **(C)** The amplification products of the wild-type and variant minigene constructs (c.3990G > A) were separated by 2% agarose gel electrophoresis. The lengths of the two bands were the same. **(D)** Direct sequencing showed that the two transcripts were identical except for the mutant nucleotide (c.3990G > A). **(E)** The amplification products of the wild-type and variant minigene constructs (c.4766C > T) were separated by 2% agarose gel electrophoresis. The lengths of the two bands were the same. **(F)** Direct sequencing showed that the two transcripts were identical except for the mutant nucleotide position (c.4766C > T). WT, wild-type; MT, mutant-type; F, forward primer; R, reverse primer.

Figure 3A shows that the length of the wild-type minigene transcription product was equal to that of the variant (c.3566G > A in *COL4A3*) after PCR amplification and agarose gel electrophoresis. Direct sequencing indicated that the two transcripts were all 320 bp and made up of partial exon 41, exon 42 and partial exon 43 (Figure 3B). Only the first nucleotide of exon 42 was discrepant between the wild-type and mutant-type transcripts, which suggested that the variant was missense, not compatible with the prediction of Human Splicing Finder and MaxEntScan. The variant c.4793T > G was the same as patient 1 and reclassified as “pathogenic”; thus, the variant c.3566G > A *in trans* was re-evaluated as “likely pathogenic” (PM2, PM3, PP3 and PP4). Autosomal recessive Alport syndrome was diagnosed in patient 2 according to the clinical manifestations, renal biopsy results, family history of haematuria, and genetic testing results. The recurrence risk of her siblings was 25%.

The length of the wild-type minigene transcription product was equal to that of the variant (c.3990G > A in *COL4A4*) after PCR amplification and agarose gel electrophoresis (Figure 3C). Direct sequencing indicated that the two transcripts were all 229 bp and contained part of exons 41 and 42 (Figure 3D). Only the mutant-type transcript harboured the variant c.3990G > A, which suggested that the variant was synonymous and not compatible with the prediction of Human Splicing Finder and NetGene2. Considering the clinical manifestations and minigene assay results, it was believed that c.3990G > A was not pathogenic, and TBMN was highly suspected in patient 4, but long-term follow-up was needed to confirm the diagnosis.

The length of the wild-type minigene transcription product was equal to that of the variant (c.4766C > T in *COL4A4*) after PCR amplification and agarose gel electrophoresis (Figure 3E). Direct sequencing showed that the two transcripts were all 592 bp and contained partial exon 46, exon 47 and partial 48 (Figure 3F). Only the mutant-type transcript harboured the variant c.4766C > T, which suggested that the variant was missense and not compatible with the prediction of Human Splicing Finder and MaxEntScan. The pathogenicity re-evaluation had not been changed. Considering the clinical manifestations, renal biopsy and minigene assay results, Alport syndrome could be diagnosed in patient 5. However, the inheritance pattern could not be determined since her family members had no haematuria and we lacked parental DNA samples.

## Discussion

In this study, we assessed the pathogenicity of five *COL4A3/COL4A4* unclassified presumed missense and synonymous variants that may affect splicing as predicted by the *in silico* tool Human Splicing Finder. It was confirmed by the minigene assay that the *COL4A3* missense variant p. (Leu1598Arg) and synonymous variant p. (Thr255Thr) theoretically speculated based on the nucleotide substitutions at the genomic DNA level may be splicing variants. Similar phenomena had been observed in *COL4A5* gene using *in vitro* minigene analysis combined with or without analysis of patient-derived RNA from urine sediments and peripheral blood leukocytes (9, 11). These pathogenic variants cannot be accurately evaluated based on the NGS results of genomic DNA alone if mRNA analysis is not performed. In addition, it has not yet been reported that reclassification of single base variants in the *COL4A3* coding region based on functional experimental results.

Splicing is catalysed by a dynamic spliceosome, which is comprised of five different small nuclear ribonucleoproteins (snRNPs). snRNPs are mainly synthesised by five small nuclear RNAs (snRNAs) named U1, U2, U4, U5, and U6 due to their uracil richness. They can accurately recognise5′ splice sites, 3′ splice sites and branch points, thereby catalysing the premRNA splicing process (12). The *cis*-regulatory elements comprise exon splicing enhancers (ESEs), intron splicing enhancers (ISEs), exon splicing silencers (ESSs) and intron splicing silencers (ISSs) located in either exons or introns. These *cis*-splicing regulatory elements work by recruiting *trans*-acting factors to activate or suppress splice site recognition or spliceosome assembly (13, 14). Therefore, variants in the genome could affect the splicing process by repressing 5′ or 3′ splice sites, thus generating new splice sites, or having an impact on recruiting *trans*-acting factors.

At present, 619 *COL4A3/COL4A4* variants have been reported, of which splicing variants account for ~13.6% (refer to the human gene mutation database, HGMD), and most of these variants are located at typical splice sites, namely highly conserved 5′ donor or 3′ acceptor sites (↓GT or AG↓). Studies have shown that 15–60% of human genetic diseases are caused by splicing variants (15), and it has been speculated that the true proportion of splicing variants in *COL4A3/COL4A4* may be underestimated. Daga et al. found that a deep intronic variant in *COL4A4* could alter splicing products by analysing RNA extracted from Alport patient's urine-derived podocyte lineage cells (16). In our previous study, atypical intronic variants were detected in 10.7% (3/28) of patients with X-linked Alport syndrome by analysing the cDNA of skin fibroblasts (7), and deep intronic splicing variants in Alport syndrome causative genes could be detected by the analysis of patient-derived RNA (17). Jourdy et al. found that synonymous variants in *F8* could lead to new transcripts by creating new splicing sites (18). RNA analysis of peripheral blood leukocytes demonstrated that missense variant p. (R919G) in *ATP7B* causing Wilson's disease could lead to exon skipping (3). Given that deep intronic, missense and synonymous variations may be cryptic splice sites, it is important to re-evaluate the pathogenicity of such variants using transcript analysis. In this study, using an *in vitro* minigene assay, it was shown that *COL4A3* variants c.4793T > G [p. (Leu1598Arg)] and c.765G > A [p. (Thr255Thr)] had effects on pre-mRNA splicing, providing supporting evidence for reclassification of their pathogenicity. Meanwhile, two other variants *in trans* with these variants were re-evaluated as likely pathogenic, allowing for accurate genetic diagnosis and genetic counselling for patients 1 and 3.

Many bioinformatics tools have been used to predict splicing variants. Among them, Human Splicing Finder has been widely used for evaluating variant effects on 5′ splice sites, 3′ splice sites, enhancers, silencers, and branch points (19). However, no splice site prediction tool can be 100% accurate. It was suggested to using multiple *in silico* tools to pinpoint unclassified variants worthy of transcript analyses (20). Nonetheless, except *COL4A3* variant c.765G > A [p. (Thr255Thr)], no splicing abnormalities were observed for the variants (c.3566G > A in COL4A3 and c.3990G > A and c.4766C > T in COL4A4) with a splicing defect predicted using at least two splice site prediction tools. Additionally, *in vitro* minigene assay analysis of *DYSF* missense and intronic variants showed that 41% of the tested variants were correctly predicted by Human Splicing Finder (6). In the present study, a false-positive prediction was observed in three of five variants, in accordance with the previous report (6). Therefore, in a molecular diagnostic setting, it is essential to compare *in silico* splicing predictions and transcript analysis results. The minigene assay is a popular approach to examine whether a splicing variant affects splicing of the neighbouring exon (21, 22), and it provides good agreement with experiments using patient-derived RNA (23, 24). Given the unavailability of specimens from our patients, such as urine or kidney tissue, and our previous study showed that *COL4A3/COL4A4* variants detected in mRNA isolated from Epstein Barr virus-transfected lymphocytes were not always confirmed at the genomic DNA level (data not reported), we used a minigene assay to analyse the consequences of the selected variants. Our study showed that the corresponding transcripts were obtained successfully, and as described above, two predictions were confirmed, which indicated that it was possible and feasible to investigate the effect of *COL4A3/COL4A4* variants on splicing by the minigene assay.

It is worth discussing the interpretation of the results of the minigene assay. The alternative transcripts, which are revealed by using different vectors and transfecting cell lines, are slightly different from those found by analysing RNA extracted from the peripheral blood (25, 26). This phenomenon may be due to differences in the expression of splicing factors or splicing regulatory factors in different cells. In addition, it may be difficult to evaluate genotype-phenotype correlations when the splicing variants change the relative proportion of different transcripts, and the minigene assay cannot precisely simulate the tissue/cell environment in which the gene is normally expressed. For example, minigene assay analysis demonstrated that the variant c.388 + 5G > A in haemophilia A causative gene *F8* leads to two transcripts: one to an abnormal transcript with skipping of the associated exon and one corresponding to the wild-type transcript, which was inconsistent with the patient's severe phenotype (18). In this study, we showed that *COL4A3* variant c. 4793T > G led to the production of a transcript lacking exon 51, whereas the wild-type minigene recombinant clone caused the expression of two transcripts: one containing exon 51 and another without exon 51. It has been previously shown that there are three distinct *COL4A3* transcripts in foetal (12 weeks) and adult kidney tissues: alternative transcript I containing exons 48–52; alternative transcript II without exon 49; and alternative transcript III without exon 51 (27). Variations could be seen in the expression of these isoforms: alternative transcript I was dominant and accounted for more than 70%, and the relative expression of alternative transcript III in foetal kidney (5.5%) was higher than that in the adult kidney (4.5%), which suggested a slight change in alternative splicing along with organ development. Therefore, due to the loss of the full-length transcript, the *COL4A3* variant c.4793T > G was considered a splicing variant. Three patients harbouring this heterozygous variant plus another heterozygous *COL4A3* nonsense variant p. (Gly709Ter), intronic splicing variant c. 3752-511_3955 + 576 del, or missense variant p. (Gly1155Asp) demonstrated the typical ultrastructural basket-weave change in the GBM or a thin GBM and an absence of type IV collagen α5 chain in the GBM and only normal immunostaining of Bowman's capsule (typical immunofluorescence staining pattern for autosomal recessive Alport syndrome) (28), suggesting these *COL4A3* variants affect type IV collagen α3 chain production.

There were some limitations in this study. First, only unclassified missense and synonymous variants in *COL4A3/COL4A4* were screened for splicing analysis. Some frameshifts (29) or nonsense variants (30) may also interfere with splicing and need to be identified. Second, only HEK293T cells were employed to assess the impact on splicing of selected exonic substitutions detected in the patients without immunostaining for type IV collagen α5 chain in renal tissues, and the samples from our patients such as urine or kidney specimen which specific expresses type IV collagen α3 and α4 chains are unavailable, to avoid possible false-negative results, at least one other human cell line, such as HeLa cells, should be used to verify the results from HEK293T cells. Third, Horinouchi et al. showed different nucleotide changes affecting the same codon may or may not affect splicing of an exon using minigenes (11), whereas there were no previously published pathogenic variants at the same codons for the variants in this study, we could not compare the effect on RNA splicing.

## Conclusion

For the first time, we demonstrated that the presumed missense variant p. Leu1598Arg and synonymous variant p. Thr255Thr in *COL4A3* affected pre-mRNA splicing, which extends this gene mutational spectrum and highlights the importance of transcript analysis of exonic sequence variants in the gene to assist with molecular diagnosis and genetic counselling. Meanwhile, we showed that the accuracy of *in silico* predictive tools for splicing variant prediction was limited.

## Data Availability Statement

The original contributions presented in the study are publicly available. This data can be found here: https://www.ncbi.nlm.nih.gov/genbank/, OL998453-OL998462.

## Ethics Statement

The studies involving human participants were reviewed and approved by Ethical Committee of Peking University First Hospital. Written informed consent from the participants' legal guardian/next of kin was not required to participate in this study in accordance with the national legislation and the institutional requirements.

## Author Contributions

HD and FW designed the research study and wrote the manuscript. HD performed the research. HD and YZ analysed the data. JD provided help and advice on designing the research and writing the manuscript. All authors contributed to editorial changes in the manuscript. All authors read and approved the final manuscript.

## Funding

This work was supported by National Nature Science Foundation (81070545), Beijing Nature Science Foundation (7102148), National Key Research and Development Program of China, the registry study of rare diseases in children (No. 2016YFC0901505), and Beijing Key Laboratory of Molecular Diagnosis and Study on Pediatric Genetic Diseases (BZ0317).

## Conflict of Interest

The authors declare that the research was conducted in the absence of any commercial or financial relationships that could be construed as a potential conflict of interest.

## Publisher's Note

All claims expressed in this article are solely those of the authors and do not necessarily represent those of their affiliated organizations, or those of the publisher, the editors and the reviewers. Any product that may be evaluated in this article, or claim that may be made by its manufacturer, is not guaranteed or endorsed by the publisher.
